# Effect of periapical surgery on oral health-related quality of life in the first postoperative week using the Dutch version of Oral Health Impact Profile-14

**DOI:** 10.1007/s10006-021-00954-y

**Published:** 2021-03-24

**Authors:** Jacco G. Tuk, Jerome A. Lindeboom, Arjen J. van Wijk

**Affiliations:** 1 Departments of Oral and Maxillofacial Surgery, Amsterdam University Medical Center Amsterdam and Amstelland Hospital Amstelveen, Meibergdreef 9 , 1105 AZ Amsterdam, The Netherlands; 2 Department of Social Dentistry, Academic Centre for Dentistry Amsterdam (ACTA), University of Amsterdam and Vrije Universiteit Amsterdam, Amsterdam, The Netherlands

**Keywords:** Periapical surgery, Pain, OHRQoL

## Abstract

**Objective:**

To evaluate whether periapical surgery affects oral health-related quality of life (OHRQoL) within the first postoperative week.

**Study design:**

The primary outcomes in 133 patients (54 men, 79 women; mean age 50.8 years) undergoing periapical surgery were the Oral Health Impact Profile-14 (OHIP-14) score and postoperative sequelae, including pain, analgesic intake, swelling, limited mouth opening, chewing difficulties, and postoperative infection.

**Results:**

We found a significant effect on OHIP-14, pain, and analgesics, which decreased throughout the week. We found no significant differences in mean OHIP-14, pain scores, or analgesic use for gender, medical history, surgical flaps, operation time, or location of the operated teeth. Younger patients had a higher OHIP-14 score in the first 2 days after surgery and more pain on the first postoperative day. Women experienced more pain during the first 3 days. Smokers had a higher OHIP-14 score on the first postoperative day and greater pain during the first 3 days compared to non-smokers.

**Conclusion:**

We identified a low incidence of pain and reduced OHRQoL following periapical surgery. The postoperative reduction in OHRQoL and pain were of short duration, with maximum intensity in the early postoperative period and rapidly decreasing with time.

## Introduction

Periapical surgery is a therapeutic surgical procedure to treat teeth with periapical inflammation, particularly when orthograde retreatment is problematic or fails to lead to regression of the apical pathology [1, 2]. As with any surgery, periapical surgery causes tissue damage and can have both a local and systemic impact that deteriorates the patient’s quality of life (QoL). There has been little emphasis on immediate postoperative outcomes, such as pain, swelling, and the patient’s well-being after periapical surgery, but the number of studies evaluating the influence on QoL during the period following endodontic surgery is growing [3–17]. In the decision-making process regarding endodontic surgery, clinicians need to consider patients’ postoperative discomfort. Pain and swelling are common following periapical surgery, but postoperative pain is reported to be of short duration, with a maximum intensity in the first 48 h [3–13]. Routine daily activities, function, and loss of work are reported to be only moderately impaired [14]. Several studies have investigated additional interventions to ameliorate the effect of periapical surgery on postoperative pain and QoL [9, 15–17]. The use of corticosteroids has been reported as a pain relief measure in periapical surgery [9], although another study failed to find an effect of submucosal injection of 4 mg dexamethasone on pain, bruising, and wound healing [16]. Conflicting outcomes have also been reported for the use of platelet concentrates in periapical surgery. Del Fabbro et al. [15] found a significant beneficial effect of adjunct platelet concentrate on postoperative QoL, whereas a recent study evaluating the impact of adjunct leukocyte and platelet-rich-fibrin on QoL after periapical surgery found no significant improvement during the first postoperative week [15–17].

The aim of the present study was to investigate whether periapical surgery affects oral health-related quality of life (OHRQoL) during the first postoperative week. Patients were surveyed using the Oral Health Impact Profile-14 (OHIP-14) questionnaire. In addition, we assessed postoperative pain, analgesic intake, and infection in the first postoperative week.

## Materials and methods

### Patient selection

Patients referred by their dentists for periapical surgery at the Department of Oral and Maxillofacial Surgery of Amstelland Hospital in Amstelveen, the Netherlands, during 2017 and 2018 were eligible for inclusion in this prospective study. The study was reviewed and approved by the institutional Medical Ethics Committee of the Academic Medical Centre of the University of Amsterdam and conducted in accordance with Good Clinical Practice and the Declaration of Helsinki, as amended in Somerset West, Republic of South Africa, in 1996. Patients were fully informed about the surgical procedure, postoperative care, follow-up examinations, and alternative treatment options. Each patient was informed that they could withdraw from the study at any time without consequences regarding their treatment.

### Inclusion and exclusion criteria

Patients with apical periodontitis in a root canal-treated tooth were included in this study. Asymptomatic patients aged ≥ 18 years and in good health (American Society of Anesthesiologists (ASA) I or II) who were willing to participate and able to read, understand, and answer the questionnaire were considered for inclusion if they had periapical periodontitis with no possibility of root canal retreatment or the ability to achieve better results with a nonsurgical approach. Patients underwent a clinical and radiographic examination, and a panoramic radiograph and periapical radiograph were taken. The tooth to be treated had to have an adequate final restoration without clinical evidence of coronal leakage. No acute symptoms were present, and the diameter of the periapical lesions had to be < 10 mm as measured on the periapical radiograph.

Exclusion criteria were other causes related to root pathology other than apical re-infection, such as root fractures, teeth with an inadequate coronal restoration, perforations and bone loss (periodontal pockets deeper than 7 mm), and defects of the buccal and lingual cortical bone, as suggested by Zuolo et al. [18]. Other exclusion criteria were antibiotic prophylaxis, a history of a recent and/or symptomatic peptic ulcer, antiplatelet or anticoagulant therapy, pregnancy or lactation, recent (< 15 days) acute local infection before surgery, previous radiation therapy to the maxillofacial region, or lack of consent to undergo the procedure or participate in the study.

### Surgery

The surgery was performed by two surgeons (JT and JL). Patients received local anesthesia with 40 mg of articaine/hydrochloride and 0.01 mg epinephrine (Ultracain D-S Forte, Sanofi-Aventis Netherlands BV, Gouda, the Netherlands). The surgical technique consisted of a midlevel, rectangular or triangular, full-thickness mucoperiosteal flap. The surgical flap was reflected, and bone removed by a round burr with continuous sterile distilled water irrigation to expose the root apex. After debridement of the pathological tissue, the root was resected approximately 3 mm from the apex using a cylinder burr with minimal or no bevel. Using glasses with 5.0 magnification loupes and a PureLight Headlamp with 140 mm spot size (SL Company, London, UK), the root end was prepared using ultrasound to a 2–3-mm depth with ultrasonic retro-tips (Mectron S.p.A., Carasco, Italy). Intermediate Restorative Material (IRM, Dentsply, Konstanz, Germany) was placed into a dried cavity after adequate hemostasis. Before wound closure, the bone cavity was cleaned with 10 ml of 0.9% NaCl solution (B Braun, Melsungen, Germany). The wound was closed by re-approximating the soft tissue to the original position and sutured with Vicryl 4/0 (Johnson and Johnson; Somerville, NJ) before taking final radiographs.

### Postoperative instructions

After surgery, patients were given verbal and written instructions, including information about swelling, using an ice pack for cooling the cheek to reduce swelling and pain relief, avoiding mouth rinsing and spitting, practicing caution when eating and drinking hot food and beverages, and avoiding physical activities. Patients < 50 years of age with an ASA I classification were prescribed 600 mg ibuprofen (Brufen; Abbot BV, Hoofddorp, the Netherlands) three times a day postoperatively, whereas patients ≥ 50 years old or with an ASA II classification were prescribed 1000 mg paracetamol 3–4 per day postoperatively. No antibiotics were prescribed. The day after surgery, patients began using a 0.12% aqueous chlorhexidine mouth rinse twice a day for 1 min for 7 days. Patients were informed to contact the surgeon if they experienced severe pain, swelling, fever, bleeding, or any concerns after surgery.

### Follow-up

One week after surgery, patients were examined by an independent assessor to assess surgical site wound healing and to check for wound infection. Remaining resorbable sutures were removed. Infection was defined as the presence of purulent discharge and/or excessive swelling with fluctuation, with or without pain; presence of a local abscess; or onset of facial or cervical cellulitis plus other signs suggesting infection, such as pain, increased heat, temperature, erythema, and/or fever [19]. In patients in whom infection was diagnosed, drainage was followed by a 5-day course of amoxicillin three times a day. The number of postoperative visits, type and amount of analgesic, type and dosage of antibiotic, and interventions were documented. The completed OHIP-14 questionnaires and pain scores were collected.

### Outcome measurements

The primary outcome measures were the OHIP-14 questionnaire and pain score based on the numeric rating scale (NRS). Each patient was asked to complete a questionnaire in the first 7 days postoperatively. The questionnaire was translated into Dutch, comprising 14 questions to evaluate the OHRQoL on a 5-point scale ranging from 0 (“never”) to 4 (“very often”) [20, 21]. Higher scores on the OHIP-14 (range 0–56) indicated a worse OHRQoL. The questionnaire was supplemented with additional questions on analgesic use and postoperative symptoms, such as limited mouth opening, limited chewing, and swelling. The patients were asked to complete the daily OHIP-14 questionnaire, to evaluate pain and analgesic intake at the end of each day. Pain assessment was measured by rating pain intensity with an 11-point NRS, which ranged from 0 (no pain) to 10 (worst possible pain). The daily analgesic intake was self-reported, by filling in the number of used painkillers on each postoperative day.

### Data management

Data were collected and imported into a database. Variables included patient age, gender, medical history, and smoking habits. Age at surgery was computed in years as the difference between the date of operation and the patient’s date of birth. Furthermore, the location of the treated tooth, surgical flap design, and operation times were recorded.

### Statistical analysis

Data were analyzed using SPSS version 25 (SPSS Inc., Chicago, IL, USA). Significance was set at *α* = 0.05. To obtain the overall mean OHIP-NL14 score, all 14 questions were averaged for each day, and this score was used to compare changes over time and between groups. Repeated measures ANOVA within subjects was performed to assess the change over time (day 1–7). Additional analyses were conducted to determine the relationship between OHRQoL and the other study variables (age, gender, smoking, ASA classification, and tooth position) over time by means of univariate analysis of variance. Between-group comparisons were performed by means of independent *t*-tests.

## Results

A total of 133 patients (54 (40.6%) males and 79 (59.4%) females) participated in this study, and all questionnaires were included in the study. The mean patient age was 50.8 years (SD 14.7) for the whole population, 50.7 years (SD 14.8) for the males, and 51 years (SD 14.7) for the females. Surgery was performed in 22 maxillary anterior teeth (16.5%), 29 maxillary premolars (21.8%), 37 maxillary molars (27.8%), 3 mandibular anterior teeth (2.3%), 5 mandibular premolars (3.8%), and 37 mandibular molars (27.8%).

### OHIP-14 scores

**Fig. 1:**
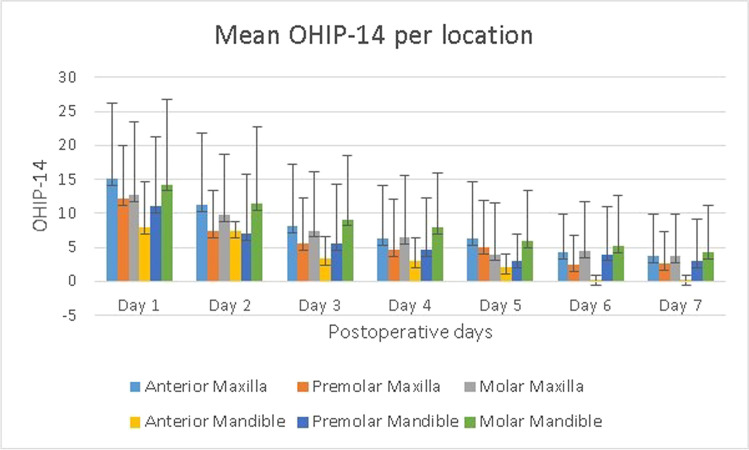
Mean OHIP-14 score per location during the 1st postoperative week. Error bars indicate SD

Of the 133 returned questionnaires, the mean overall OHIP-14 score was determined for postoperative days 1 to 7 (Table 1). Repeated measures ANOVA was used to analyze the mean overall OHIP-14 scores collected each day during the first postoperative week, indicating a significant effect for the repeated measurements (F(6, 792) = 72.8, *p* < 0.001). Subsequent pairwise comparisons indicated that the mean OHIP-14 scores decreased significantly throughout the week. Only the mean scores from day 5 and day 6 did not differ significantly (*p* = 0.11), whereas all the mean scores on the other days differed significantly from each other (*p* < 0.05). No significant differences in mean OHIP-14 scores were found for gender, ASA score, surgical flaps, or operation time. Smokers had a significantly higher OHIP-14 score on the first postoperative day than non-smokers. Patients who had a postoperative infection had a significantly higher OHIP-14 score on the fifth postoperative day. Younger patients had a significantly higher OHIP-14 score on the first 2 postoperative days compared to the older patient groups. Figure 1 shows the mean OHIP-14 scores per location. No significant interaction effect between time and OHIP-14 score was found for anterior teeth, premolars, and molars in the upper or lower jaw. Comparing the second molar region with the other locations, no significant differences were found during the week for the mean OHIP-14 scores during the first 3 days (day 1, *p* = 0.84; day 2, *p* = 0.34; day 3, *p* = 0.27).

**Table 1:** OHIP-14 scores on postoperative days 1–7

Group	Sample	Mean SD	Day 1	Day 2	Day 3	Day 4	Day 5	Day 6	Day 7
Men	54	Mean	11.48	8.26	6.11	5.44	4.83	3.91	3.43
		SD	10.31	9.34	8.59	7.71	6.22	6.63	6.37
Women	79	Mean	14.47	10.86	8.35	6.97	5.20	4.28	3.61
		SD	10.61	9.17	8.31	8.19	7.97	6.21	5.80
ASA I	88	Mean	13.63	10.01	7.52	6.75	4.78	4.43	3.97
		SD	10.93	9.21	8.37	8.10	6.94	6.49	6.62
ASA II	45	Mean	12.53	9.40	7.29	5.58	5.58	3.53	2.69
		SD	9.86	9.54	8.75	7.84	7.99	6.14	4.55
Smokers	17	Mean	18.59*	11.76	8.12	5.88	4.82	3.35	2.47
		SD	11.48	8.58	7.60	5.70	6.12	4.85	4.14
Non-smokers	116	Mean	12.47*	9.52	7.34	6.42	5.09	4.24	3.69
		SD	10.23	9.39	8.61	8.31	7.47	6.57	6.24
Age 18–25 yrs	5	Mean	19.20*	12.00*	9.00	10.00	0.20	2.40	1.60
		SD	12.46	7.81	5.61	8.28	0.45	2.61	1.82
Age 26–45 yrs	45	Mean	17.60*	12.78*	9.91	7.91	6.67	5.71	4.89
		SD	11.31	10.95	9.84	8.55	7.47	7.48	7.46
Age 46–65 yrs	61	Mean	11.13*	8.64*	6.18	4.90	4.18	3.05	2.75
		SD	9.24	7.90	6.86	5.99	6.87	4.95	4.85
Age > 65 yrs	22	Mean	8.91*	6.55*	5.55	6.36	5.27	4.27	3.36
		SD	8.74	8.12	9.22	10.94	8.29	7.52	6.03
Postop infection	7	Mean	15.00	14.14	13.29	11.57	10.43*	8.00	7.43
		SD	14.40	14.37	11.60	6.06	3.51	4.20	5.68
No postop infection	126	Mean	13.16	9.56	7.12	6.60	4.75*	3.91	3.32
		SD	10.37	8.95	8.20	8.00	7.34	6.41	5.98
Quadr. flap	63	Mean	13.65	10.17	8.06	6.94	5.19	4.67	4.02
		SD	12.01	10.70	9.31	7.99	7.21	7.06	6.80
Triang. flap	60	Mean	12.23	8.85	6.38	5.32	4.32	3.25	2.70
		SD	8.31	7.10	7.12	7.62	6.54	5.39	4.43
Midlevel flap	10	Mean	16.90	13.20	9.90	8.90	8.60	6.00	5.50
		SD	12.81	11.31	10.25	10.12	11.11	7.04	8.55
Surgery									
< 20 min	63	Mean	14.78	10.68	7.97	6.59	5.29	4.11	3.25
		SD	10.43	10.02	9.27	8.79	7.62	5.97	5.34
20–25 min	18	Mean	12.22	8.72	6.33	5.56	5.28	4.50	4.17
		SD	9.21	6.68	7.23	7.00	6.43	6.65	6.45
26–30 min	44	Mean	12.63	9.55	7.72	6.84	5.23	4.41	3.84
		SD	11.48	9.54	8.28	7.78	7.75	7.28	6.82
> 30 min	8	Mean	7.00	6.75	4.25	3.63	1.75	1.88	2.63
		SD	7.05	7.05	4.95	4.63	2.12	2.85	6.30
Overall	133	Mean	13.26	9.80	7.44	6.35	5.05	4.13	3.53
		SD	10.55	9.87	8.47	8.00	7.29	6.37	6.01

*quadr* quadrangular; *triang* triangular; *SD* standard deviation

^*^p < 0.05

### Pain scores

Repeated measures were used to determine mean scores over time for pain from postoperative days 1 to 7. Repeated measures ANOVA was used to analyze mean NRS scores collected each day during the first postoperative week (Table 2). We found a significant effect for the repeated measurements (F(6, 792) = 61.3, *p* < 0.001). Subsequent pairwise comparisons showed that the mean NRS scores decrease significantly throughout the week. Only the mean scores from day 3 and day 4 did not differ significantly (*p* = 0.15), whereas all the mean scores on the other days differed significantly from each other (*p* < 0.05).

**Table 2:** NRS pain scores for the 1st postoperative week

Group	Sample		Day 1	Day 2	Day 3	Day 4	Day 5	Day 6	Day 7
Men	54	Mean	2.50*	2.06*	1.72*	1.64	1.72	1.52	1.94
		SD	2.31	2.29	2.06	2.13	2.18	2.37	2.26
Women	79	Mean	3.75*	2.92*	2.48*	2.17	1.71	1.35	1.11
		SD	2.45	2.46	2.25	2.21	2.01	1.69	1.58
ASA I	88	Mean	3.40	2.60	2.23	2.06	1.80	1.53	1.24
		SD	2.45	2.40	2.13	2.24	2.12	2.09	1.99
ASA II	45	Mean	2.94	2.51	2.06	1.76	1.57	1.19	0.94
		SD	2.50	2.47	2.36	2.09	1.99	1.77	1.64
Smokers	17	Mean	4.76*	3.65*	3.24*	2.47	1.65	1.29	1.06
		SD	2.54	2.42	2.17	1.91	1.58	1.40	1.52
Non-smokers	116	Mean	3.03*	2.41*	2.02*	1.88	1.73	1.44	1.16
		SD	2.39	2.39	2.17	2.22	2.14	2.06	1.93
Age 18–25 yrs	5	Mean	4.80*	3.40	3.00	3.00	1.20	1.00	0.70
		SD	1.92	1.34	1.87	2.55	1.10	1.22	1.10
Age 26–45 yrs	45	Mean	3.70*	2.91	2.57	2.19	1.90	1.76	1.38
		SD	2.28	2.52	2.28	2.16	1.98	2.10	2.10
Age 46–65 yrs	61	Mean	3.22*	2.62	2.14	1.88	1.69	1.30	1.16
		SD	2.71	2.54	2.29	2.23	2.15	1.87	1.91
Age > 65 yrs	22	Mean	2.05*	1.55	1.27	1.45	1.55	1.14	0.73
		SD	1.81	1.79	1.55	2.04	2.30	2.21	1.39
Postop infection	7	Mean	2.29	2.14	2.14	2.29	3.00	2.86*	2.29
		SD	1.80	2.19	1.77	2.21	2.08	2.41	2.63
No postop infection	126	Mean	3.30	2.60	2.17	1.94	1.65	1.34*	1.08
		SD	2.49	2.44	2.22	2.19	2.06	1.94	1.82
Quadr. flap	63	Mean	3.52	2.71	2.42	2.06	1.81	1.47	1.20
		SD	2.55	2.71	2.51	2.31	2.16	2.09	2.07
Triang. flap	60	Mean	2.78	2.30	1.97	1.80	1.59	1.34	1.09
		SD	2.32	2.09	1.78	2.10	2.05	1.96	1.76
Midlevel flap	10	Mean	4.40	3.30	2.90	2.20	1.90	1.55	1.10
		SD	2.46	2.31	2.13	1.99	1.85	1.64	1.45
Surgery									
< 20 min	63	Mean	3.51	2.81	2.17	2.02	1.97	1.64	1.24
		SD	2.60	2.47	2.17	2.23	2.25	2.22	1.85
21–25 min	18	Mean	3.06	2.11	1.83	1.58	1.33	1.22	1.28
		SD	2.53	2.47	2.43	1.85	1.61	1.90	2.37
26–30 min	44	Mean	3.11	2.57	2.45	2.15	1.69	1.27	0.98
		SD	2.34	2.42	2.27	2.38	2.06	1.71	1.72
> 30 min	8	Mean	2.38	1.75	1.38	1.25	0.75	0.88	1.00
		SD	2.00	1.83	1.19	1.39	1.39	1.99	2.07
Overall	133	Mean	3.25	2.57	2.17	1.95	1.72	1.42	1.14
		SD	2.47	2.42	2.20	2.19	2.07	1.99	1.88

*quadr* quadrangular; *triang* triangular; *SD* standard deviation

^*^p < 0.05

**Fig. 2:**
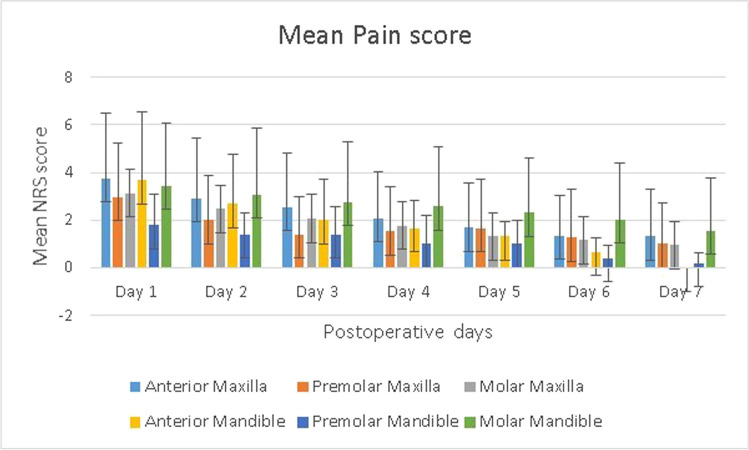
Mean numeric rating scale (NRS) pain score per location during the 1st postoperative week. Error bars indicate SD

Women and smokers experienced significantly more pain during the first 3 days. Younger patients had a higher pain score compared to older patients on the first postoperative day. We found no significant interaction effect during the first postoperative week for pain scores and ASA group, surgical flaps, location of teeth, or operation time. Comparing the second molar region with the other locations, we found no significant differences during the week for the NRS pain scores, or even during the first 3 days (day 1, *p* = 0.30; day 2, *p* = 0.32; day 3, *p* = 0.29). Figure 2 shows the pain scores versus the location of the operated teeth.

### Analgesic intake

**Fig. 3:**
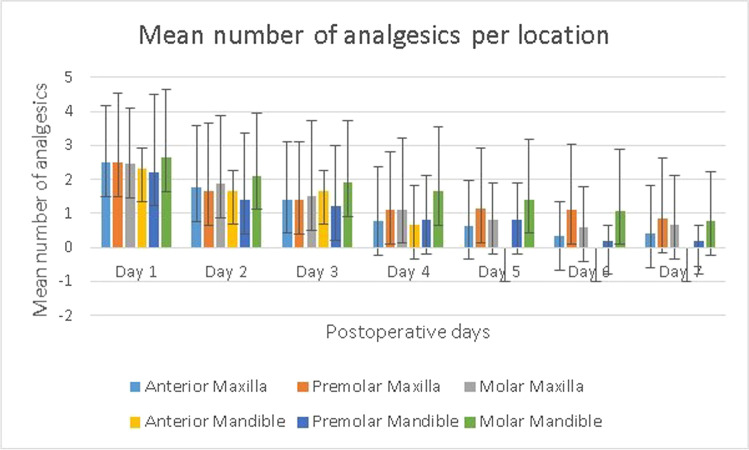
Mean analgesic consumption score per location during the 1st postoperative week. Error bars indicate SD

Repeated measures were used to determine mean scores over time for pain from postoperative days 1 to 7. Repeated measures ANOVA was used to analyze mean analgesic intake each day during the first postoperative week (Table 3). We found a significant effect for the repeated measurements (F(6, 127) = 26.8, *p* < 0.001). Subsequent pairwise comparisons show that the mean analgesic use decreased significantly throughout the week. Only the mean scores from days 4 and 5 and days 6 and 7 did not differ significantly (*p* = 1.00), whereas all of the mean scores on the other days differed significantly from each other (*p* < 0.05). We found no significant interaction effect during the first postoperative week for mean analgesic intake and gender, ASA group, smokers, surgical flaps, operation time, or location of teeth. Figure 3 shows the mean number of analgesic intake for the location of the operated teeth. On the first postoperative day, 14.3% of patients reported not using any analgesics. This percentage increased to 30.8% on day 2 and 42.1% on day 3. On the seventh day, 23.3% of the patients used analgesics.

**Table 3:** Analgesic intake during the 1st postoperative week

Group	Sample		Day 1	Day 2	Day 3	Day 4	Day 5	Day 6	Day 7
Men	54	Mean	2.24	1.58	1.25	0.87	0.85	0.77	0.62
		SD	1.82	1.74	1.53	1.41	1.45	1.64	1.51
Women	79	Mean	2.70	1.96	1.79	1.40	1.09	0.75	0.68
		SD	1.80	1.88	2.04	2.07	1.72	1.48	1.45
Smokers	17	Mean	2.53	2.47	2.12	1.12	0.47	0.47	0.53
		SD	1.70	2.15	1.93	1.69	1.18	1.18	1.50
Non-smokers	116	Mean	2.51	1.76	1.50	1.20	1.07	0.80	0.68
		SD	1.84	1.83	1.85	1.88	1.64	1.58	1.47
ASA I	88	Mean	2.40	1.85	1.61	1.27	1.03	0.86	0.69
		SD	1.77	1.80	1.89	1.94	1.56	1.56	1.38
ASA II	45	Mean	2.73	1.84	1.49	1.02	0.93	0.58	0.59
		SD	1.90	2.04	1.83	1.67	1.68	1.48	1.64
Age 18–25 yrs	5	Mean	2.60	2.00	2.50	2.50	0.50	0.10	1.20
		SD	1.95	1.00	3.32	4.24	0.87	0.22	2.68
Age 26–45 yrs	45	Mean	2.53	2.02	1.75	1.20	1.09	0.86	0.56
		SD	1.74	1.95	2.05	1.79	1.80	1.65	1.30
Age 46–65 yrs	61	Mean	2.36	1.82	1.34	0.93	0.82	0.75	0.66
		SD	1.84	1.94	1.60	1.58	1.45	1.61	1.57
Age > 65 yrs	22	Mean	2.86	1.54	1.64	1.54	1.41	0.73	0.73
		SD	1.96	1.79	1.81	1.87	1.66	1.28	1.24
Infection	7	Mean	2.71	2.29	2.00	1.43	1.14	1.29	1.00
		SD	2.21	1.60	1.63	1.40	0.90	1.50	1.73
No infection	126	Mean	2.50	1.82	1.55	1.17	0.99	0.73	0.64
		SD	1.80	1.90	1.88	1.87	1.63	1.54	1.46
Quadr. flap	63	Mean	2.48	1.79	1.55	1.20	0.93	0.73	0.65
		SD	1.87	1.92	2.13	2.04	1.57	1.56	1.45
Triang. flap	60	Mean	2.53	1.92	1.55	1.22	1.10	0.85	0.70
		SD	1.86	1.95	1.62	1.70	1.65	1.59	1.55
Midlevel flap	10	Mean	2.60	1.80	1.70	0.80	0.70	0.30	0.30
		SD	1.26	1.23	1.57	1.48	1.49	0.95	0.95
Surgery									
< 20 min	63	Mean	2.56	1.86	1.59	1.16	1.03	0.81	0.67
		SD	1.94	2.01	1.81	1.77	1.68	1.64	1.60
21–25 min	18	Mean	2.17	1.39	0.94	0.61	0.56	0.33	0.67
		SD	1.34	1.20	1.30	0.98	0.92	0.69	1.53
26–30 min	44	Mean	2.66	2.14	1.89	1.48	1.13	0.89	0.61
		SD	1.92	1.98	2.17	2.27	1.77	1.70	1.32
> 30 min	8	Mean	2.13	1.25	0.94	0.94	0.81	0.56	0.63
		SD	1.82	1.16	1.15	1.02	0.84	0.90	1.19
Overall	133	Mean	2.51	1.85	1.56	1.18	0.99	0.76	0.65
		SD	1.82	1.87	1.86	1.84	1.59	1.53	1.46

*quadr* quadrangular; *triang* triangular; *SD* standard deviation

### Postoperative swelling, mouth opening, and chewing difficulties

Tables 4 and 5 show the effect of periapical surgery on postoperative swelling, limitations in mouth opening, and chewing difficulties. Swelling was significantly different between genders on postoperative days 1 and 4, with women reporting more swelling. On the first postoperative day, more swelling was reported in the patients with an ASA I classification.

**Table 4:** Number of patients with select complications during the 1st postoperative week

Group	Sample	Day 1			Day 2			Day 3			Day 4			Day 5			Day 6			Day 7		
		S	M	C	S	M	C	S	M	C	S	M	C	S	M	C	S	M	C	S	M	C
Male	54	36*	27*	29	38	20	23*	31	16	17*	22*	16	15	20	11	11	16	7	12	13	7	10
Female	79	66*	52*	54	66	37	52*	57	35	42*	52*	25	32	36	21	28	31	17	20	24	13	18
ASA I	88	73*	57	57	72	42	51	61	39*	43	50	30	35	40	24	30	34	17	25	26	14	21
ASA II	45	29*	22	26	32	15	24	27	12*	16	24	11	12	16	8	9	13	7	7	11	6	7
Smokers	17	14	11	13	15	8	12	14	8	8	12	6	6	8	4	6	7	5	4	5	3	4
Non-smokers	116	88	68	70	89	49	63	74	43	51	62	35	41	48	28	33	40	19	28	32	17	24
Quadr flap	63	52	38	41	51	29	37	46	26	30	37	23	26	27	18	19	26	14	14	22	11	13
Triang flap	60	43	34	37	46	24	33	36	21	26	33	15	18	25	11	17	18	8	16	12	6	14
Midlev flap	10	7	7	5	7	4	5	6	4	3	4	3	3	4	3	3	3	2	2	3	3	1
OT < 20 min	63	45	36	41	46	25	35	36	19	25	32	18	21	26	12	17	20	11	17	13	10	15
OT 20–25 min	18	16	11	11	13	5	8	10	3	8	8	3	6	3	2	5	3	2	4	3	1	4
OT 26–30 Min	44	34	28	25	38	25	28	36	25	22	28	19	18	23	16	15	20	10	10	19	8	8
OT > 30 Min	8	7	4	6	7	2	4	6	4	4	6	1	2	4	2	2	4	1	1	2	1	1
Total	133	102	79	83	104	57	75	88	51	59	74	41	47	56	32	39	47	24	32	37	20	28

*S* swelling; *M* limited mouth opening; *C* chewing difficulties; *OT* operation time; *quadr* quadrangular; *triang* triangular

^*^p < 0.05

**Table 5:** Number of patients experiencing select complications during the 1st postoperative week per location

Location	Sample	Day 1			Day 2			Day 3			Day 4			Day 5			Day 6			Day 7		
		S	M	C	S	M	C	S	M	C	S	M	C	S	M	C	S	M	C	S	M	C
Anterior maxilla	22	15	14	14	16	7*	10	12	4*	6	9	4*	6	6*	2	6	3*	1	3	2*	1	2
Premolar maxilla	29	22	19	16	24	11*	15	18	8*	11	15	8*	9	9*	5	7	9*	4	8	7*	3	8
Molar maxilla	37	26	24	19	24	11*	21	23	13*	15	17	9*	13	12*	11	11	12*	7	9	7*	6	9
Anterior mandible	3	3	1	3	3	3*	2	3	3*	2	3	3*	2	3*	2	1	2*	2	1	2*	2	1
Premolar mandible	5	5	3	3	4	3*	3	3	2*	3	3	2*	3	1*	1	2	1*	1	2	0*	0	2
Molar mandible	37	31	22	24	33	22*	24	29	21*	22	27	15*	14	25*	11	12	20*	9	9	19*	8	6
Total	133	102	83	79	104	57	75	88	51	59	74	41	47	56*	32	39	47	24	32	37	20	28

*S* swelling; *M* limited mouth opening; *C* chewing difficulties

^*^Significant < 0.05

A significant difference in mouth opening was found on days 2, 3, and 4 for teeth surgically treated in the lower jaw. Postoperative swelling persisted longer in mandibular locations, especially the molars, and was significant on days 5, 6, and 7. Limitations in mouth opening were reported significantly more in females on the first postoperative day and in the ASA I group on the third postoperative day.

### Postoperative complications

Seven (5.3%) patients with a postoperative infection presented with increasing swelling at the surgical site on postoperative day 6. The abscess was drained, followed by a 5-day oral course of 500 mg amoxicillin three times a day. The patients with a postoperative infection had a significantly higher OHIP-14 score on the fifth postoperative day and a significantly higher pain score on the sixth postoperative day, which corresponded with the day that the abscess was drained (Tables 1 and 2).

## Discussion

In the present study, we assessed how periapical surgery affects postoperative OHRQoL and found the greatest effect of periapical surgery on OHRQoL and NRS pain scores during the first postoperative day, gradually decreasing through the first postoperative week. Compared to earlier studies on postoperative OHRQoL and pain after third molar surgery, periapical surgery only had a mild to moderate effect during the first postoperative week [19–21]. This finding is supported by other studies that found maximal postoperative symptoms on days 1 to 3, which then generally subsided [5, 6]. In the present study, we found no significant differences in mean OHIP-14 scores between males and females.

Postoperative pain is not uncommon following periapical surgery, and is usually of short duration, with a maximum intensity either on the day of the surgical procedure or the next day [3, 7, 9, 14]. In the present study, the mean NRS pain score was highest during the first 3 days. The mean NRS pain score was 3.25 (SD 2.47) on day 1, decreasing to 2.57 (SD 2.42) on day 2 and gradually decreasing through the week. Iqbal et al. [11] reported a mean pain score on day 1 of 3.17 (SD 2.03), and other studies have reported mean peak visual analog scale (VAS) scores of approximately 30, which is comparable to the present study [3, 4]. Garcia et al. found the highest pain score on day 2 [10].

The postoperative mean pain score is influenced by the analgesics taken by patients and, as such, does not truly reflect the real pain caused by the surgery. To obtain a real measurement of the pain after periapical surgery, patients should refrain from taking analgesics; however, as pointed out by Seymour et al., this approach would be unethical [3].

The NRS pain score exhibited a significant gender difference in the first 3 days, with women experiencing more pain, but this did not affect the OHRQoL. The mean differences in OHIP-14 score were not significantly different between males and females; however, the slightly higher pain with less impact on OHRQoL observed in women may be explained by women being better at managing pain than men [22]. Therefore, the OHIP-14 score may reflect the notion that pain did not hinder day-to-day life in women as much as it did in men [22]. Interestingly, Penarrocha et al. [8] found higher pain scores for males after periapical surgery until the third postoperative day, whereas other studies reported no significant differences in pain scores between males and females after apical surgery [3, 5, 6].

In the present study, the younger age group (< 25 years) experienced a greater effect of periapical surgery during the first 2 days and more pain during the first postoperative day. This finding is in contrast to other studies that did not find any significant effect of age on postoperative symptoms after periapical surgery [3, 5, 6, 9, 10]. However, Iqbal et al. found more postoperative discomfort in younger patients [11].

In the present study, ibuprofen was used as an analgesic in younger patients and paracetamol in the ASA II group and older patients. No significant differences were found in the use of analgesics between gender, age groups, smokers or non-smokers, flap design, or location. In the present study, 85.7% of the patients reported using analgesics on the first postoperative day. This decreased during the week and, on the seventh postoperative day, 23.3% of the patients used analgesics. Earlier studies reported that 63–67% of the patients took analgesics, which meant that some patients did not take them even though pain was reported [4, 7].

Surgical operation time, ASA classification, and flap design did not significantly impact OHRQoL and NRS pain scores during the first postoperative week. Seymour et al. also failed to find a correlation between operating time and postoperative pain. Studies have reported great variety in operation time, from a mean of 25 min to a time of 140 min for a single-rooted tooth [3, 8, 12, 14]. However, no significant correlation between operation time and postoperative pain and swelling were found. Penarrocha et al. found that trapezoidal flaps caused greater pain than triangular flaps, particularly in the first 2 days [8].

We found that smokers had a significantly higher OHIP-14 score on the first postoperative day than non-smokers. In addition, smokers experienced significantly more pain during the first 3 days. Garcia et al. also found that smokers experienced greater pain throughout almost the entire first postoperative week [10].

In the present study, the operation site had no significant influence on postoperative OHRQoL or pain. This finding is in agreement with other studies [5, 6, 8, 12, 14]. One would expect more postoperative discomfort after periapical surgery in second molars, but we found no significant effect in regard to the postoperative OHRQoL or pain scores. Other studies have found greater pain after periapical surgery of maxillary anterior teeth [11], molars [9], or the lower incisors and canines [8].

Swelling is common following surgical periapical treatment. In the present study, swelling was significantly different between genders on postoperative days 1 and 4, with women reporting more swelling. Postoperative swelling persisted longer in mandibular locations and was significant on days 5, 6, and 7. Previous reports found that the maximum swelling is experienced on the first postoperative day [4, 11] and patients were more likely to experience swelling than pain [11]. Garcia et al. [10] reported that 40.3% of their patients had no or only mild postoperative swelling on the first postoperative day, whereas Tsesis et al. [5] found that 64.7% of their patients did not report any swelling; however, patients in that study received dexamethasone, which influences the postoperative outcome with regard to swelling. We found that limitations in mouth opening were significantly more common in females on the first postoperative day and in the ASA I group on the third postoperative day. A significant difference in mouth opening was also found on days 2, 3, and 4 for teeth surgically treated in the lower jaw. Swelling, chewing, and phonetic impairment were the worst 1 and 2 days after surgery [8, 14].

Several earlier studies used some form of antibiotic prophylaxis for periapical surgical procedures [9, 11, 12, 17]. In the present study, however, no antibiotics were prescribed. A previous randomized double-blind placebo-controlled trial comparing oral placebo and a preoperative dose of 600 mg clindamycin in 256 patients [23] reported an infection rate of 1.6% in the antibiotic prophylaxis group versus 3.2% in the placebo group. In the present study, 7 (5.3%) cases of postoperative infection occurred, which were treated with drainage and a 5-day course of amoxicillin. Patients with a postoperative infection had a significantly higher OHIP-14 score on day 5 and more pain on day 6.

This study has some limitations. First, only asymptomatic cases were included; therefore, no conclusions can be drawn about the impact on OHRQoL in cases of acute periapical surgery. Second, we did not use an operating microscope in the periapical procedure. An operating microscope is used for optimal identification of root canals, fractures, and isthmuses [17], and some studies have reported that the use of microsurgical techniques is associated with less postoperative pain [1, 5, 6]. Magnification was used in the present study, but the × 5 magnification with the surgical loupes does not compare to visualization of 16 to 32 times as with the microscope. Although an earlier study did find that patients undergoing periapical surgery using a surgical microscope recovered sooner with respect to pain, no significant difference was found in postoperative swelling [1]. A disadvantage of performing periapical surgery with a microscope is that the procedure takes twice as long. Tsesis et al. [5] reported an average operating time of 20 min for periapical surgery without a microscope versus 40 min for periapical surgery using a microscope [6]. Moreover, in that study, the patients from the group operated on using a microscope experienced more difficulty in mouth opening, mastication, and the ability to speak during the first 2 days after surgery. In addition, no significant differences in pain were observed in those first 2 days. The differences in pain became clear starting with the fourth postoperative day, but the mean pain scores were ~ 2 on a 5-point scale. In contrast, in the present study, the mean pain scores were ≤ 2 on an 11-point NRS.

Another limitation of the present study is that, although the OHIP-14 is a reliable and validated tool to measure OHRQoL, data acquired from the patients are self-reported. The usual disadvantage with questionnaires is that data acquisition is subjective, and the data cannot be controlled. As such, some bias may be present [19, 24]. Facial swelling as such was not measured but reported on the OHIP-14 questionnaire, so the OHIP-14 scores were used to subjectively assess postoperative swelling. Objective methods for assessing the degree of postoperative swelling are more accurate than the estimations made by patients themselves, but as stated by Happonen et al. [25], there is no real objective way to assess the degree of intraoral swelling, which is experienced by the patients as being at least as unpleasant as extraoral swelling. Moreover, the amount of postoperative swelling is inter-individually different and the absence of a control group in the present study makes it difficult to draw a significant conclusion.

## Conclusions

We identified a low incidence of postoperative pain and reduced OHRQoL following periapical surgical treatment. The postoperative reduction in OHRQoL and pain were of short duration, with maximum intensity in the early postoperative period and decreasing with time.
